# Study on the diagnostic efficacy of the computerized tomography attenuation value of renal pelvis urine in true bacteriuria secondary to renal and ureteral stones

**DOI:** 10.1080/0886022X.2025.2561786

**Published:** 2025-09-21

**Authors:** Bowen Chen, Shuai Liu, Hongming Zhou, Yingchun Ren, Tiancheng Xie, Yunze Dong, Yucheng Gao, Xiao Xu, Yanhua Chen, Ding Liu, Hao Chen, Xiangcheng Zhan, Xudong Yao, Yunfei Xu

**Affiliations:** a Department of Urology, Shanghai Tenth People’s Hospital, Tongji University School of Medicine, Shanghai, China; b Tongji University School of Medicine, Shanghai, China

**Keywords:** CT value, urinary tract stones, urinary tract infections, perioperative management, hydronephrosis, urinary tract obstruction

## Abstract

To explore the diagnostic efficacy of the computed tomography (CT) attenuation value of renal pelvic urine in true bacteriuria secondary to renal and ureteral stones, and to construct a nomogram prediction model. This retrospective study analyzed data from patients with renal and ureteral stones. The participants were categorized into an infection group (*n* = 41) and a control group (*n* = 208) based on their urine culture. Risk factors were identified via logistic regression, and ROC analysis was used to evaluate the diagnostic performance. A risk prediction nomogram model was constructed and verified for risk factors. The infection group exhibited higher renal pelvis urine CT values (13.41 ± 5.11 vs. controls, *P < 0.0001*), with optimal diagnostic accuracy [area under the curve (AUC) = 0.867]. Logistic regression analysis showed that advanced age, positive urinary leukocyte esterase (LE) (+) levels, and increased CT values of the renal pelvis urine were risk factors. The AUC of the nomogram model was 0.94. The calibration curve and clinical decision curve analysis verified that this model had a good predictive performance. The CT value of renal pelvic urine can be used to predict the risk of bacteriuria secondary to renal and ureteral stones. An average CT value of renal pelvis urine >7.25 HU, age >57.5 years, and positive urine LE (≥1+) are risk factors. The nomogram prediction model incorporating these factors allowed for the rapid assessment of patients with urinary tract stones, demonstrating high predictive accuracy and significant clinical value.

## 1. Introduction

The diagnosis and management of urinary tract stones complicated by infections are important parts of the perioperative management of surgical procedures for urinary tract stones. This condition mainly arises from two factors. First, urinary tract stones themselves serve as the infection source, for example, struvite stones result from the long-term colonization of urease-producing bacteria (*Proteus*, *Klebsiella*, and *Staphylococcus*) [1], and stone impaction induces renal pelvic outflow tract obstruction, leading to impaired renal pelvis urine excretion and reduced metabolite clearance capacity, resulting in metabolite accumulation in the kidneys and urinary tract infections (UTIs). Uncontrolled UTIs are contraindications to surgical procedures for stones. If UTIs are not effectively controlled before surgical procedures, bacteria may enter the bloodstream via surgical wounds or increase intraoperative renal pelvic pressure, potentially leading to urosepsis and life-threatening complications [2]. Critically, complete urinary tract obstruction may prevent infected urine from reaching the bladder, resulting in false-negative cultures and delayed diagnosis. It is noteworthy that patients can rapidly progress from asymptomatic bacteriuria to urosepsis within a short period, and massive destruction of renal tissues complicated by acute kidney injury may result in a significantly higher mortality rate in such patients than in those with sepsis alone [3]. International guidelines recommend early standardized UTI treatment and urine culture screening for patients scheduled to undergo either extracorporeal shock wave lithotripsy or endoluminal procedures [4]. However, the limitations of urine cultures (poor timeliness and false negatives due to obstruction) highlight the need for rapid diagnostic alternatives.

Non-contrast-enhanced computed tomography (CT) scanning, with overall diagnostic sensitivity and specificity exceeding 90% for urinary tract stones, has become the primary method for the clinical diagnosis of urolithiasis, enabling the assessment of stone size, location, number, and hydronephrosis severity. The CT attenuation value, which indicates tissue density, is mainly used to predict stone composition. The renal pelvic urine CT value refers to the numerical attenuation measurement, expressed in Hounsfield units (HU), obtained from a CT scan of the urine contained within the renal pelvis. In clinical practice, we observed that the density of renal pelvis urine under normal conditions resembles that of water (0–10 HU); however, in cases of true bacteriuria (defined as ≥10^5^ CFU/mL in catheterized urine culture without urinary symptoms) secondary to urinary tract stones, the massive bacterial proliferation, metabolite accumulation, inflammatory/pus cell aggregation as well as tissue necrosis and shedding may lead to an increase in the renal pelvis urine density and a corresponding elevation in the CT attenuation value.

CT features and established inflammatory markers are correlated with stone-associated UTIs [5]. However, the specific relationship between renal pelvic urine attenuation (HU) and true bacteriuria, including its underlying biological mechanisms and diagnostic utility (specifically, a validated HU threshold indicative of infected urine), remains unverified and insufficiently investigated in the current literature. We hypothesized that elevated renal pelvic urine HU values are predictive of true bacteriuria secondary to urinary tract stones. To test this hypothesis, we retrospectively analyzed the correlation between renal pelvic urine HU values and positive urine cultures, quantified their diagnostic accuracy, and identified an optimal diagnostic cutoff value [6].

## 2. Materials and methods

### 2.1. Case collection

After obtaining ethical approval from the Ethics Committee, 491 patients diagnosed with urinary tract stones and confirmed to have pyelonephrosis via CT urograms at Shanghai Tenth People’s Hospital between January 2024 and June 2024 were retrospectively selected. Patients were screened and categorized into control and infected groups based on urine culture results at admission. Clinical data collected during hospitalization were analyzed.

### 2.2. Inclusion and exclusion criteria

The inclusion criteria were as follows: (1) patients admitted to our department due to unilateral renal and ureteral stones between January 2024 and June 2024; (2) complete laboratory, etiological, and urinary tract CT scans performed at our hospital; (3) non-contrast-enhanced urinary tract CT scan results suggesting mild hydronephrosis or above; and (4) patients with urinary tract stones in our hospital.

**Figure 1.**
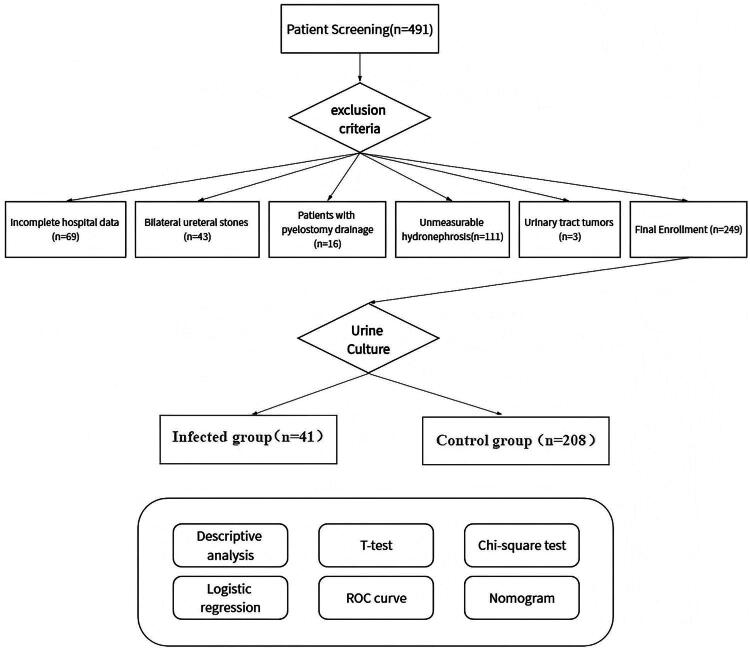
STROBE flowchart of patient selection and grouping.

The exclusion criteria were as follows: (1) patients with incomplete laboratory or CT scan results or those examined at another institution; (2) patients with a history of EWSL or bilateral urinary tract stone surgery; (3) patients with internal or external renal pelvic drainage; (4) patients without unmeasurable renal pelvic urine; and (5) patients with urological tumors or urinary system malformations.

This retrospective study exclusively enrolled treatment-naïve adults admitted for the primary surgical management of urinary tract calculi who developed radiologically confirmed hydronephrosis. All patients met stringent comorbidity-free criteria: no prior stone history/urological procedures, absence of underlying genitourinary abnormalities, exclusion of significant systemic diseases (e.g., immunocompromise) or organ dysfunction (per institutional transfer protocols), and documented freedom from recent antibiotic use/pre-existing UTIs (verified during preoperative screening) (Figure 1) [7].

### 2.3. Clinical data collection and criteria

Data on the following variables were collected:

1. General characteristics, including age, sex, and stone location.
2. Blood-associated metrics, including C-reactive protein, erythrocyte, leukocyte, platelet, hemoglobin, neutrophil, lymphocyte, monocyte, albumin, uric acid, and creatinine values. Urine-associated metrics, including leukocyte esterase (LE), nitrites, urine proteins, urine glucose, ketone bodies, urine specific gravity, pH, erythrocytes, leukocytes, and microbiological culture results.
  Urine samples were analyzed using a fully automated AVE-775 urine analyzer (AVE Science & Technology Co., Ltd.) with AVE-11a test strips. Semi-quantitative results were automatically graded and reported as: negative (−) or positive (1+/2+/3+ corresponding to +/++/+++). The analytical sensitivity (limit of detection) was 5–25 white blood cells/μL.
3. Imaging metrics, including the mean CT attenuation value of bladder urine, mean CT attenuation value of renal pelvis urine, and renal pelvis urine volume.

All non-contrast CT scans were performed on a 128-slice scanner (United Imaging uCT 760) using a standardized protocol (120 kVp, 103 mAs with automatic exposure control [AEC]), with 5 mm axial images reconstructed using the uDose iterative algorithm. Daily phantom calibration maintained HU stability (±5 HU in water). Renal pelvic and bladder urine volumes were delineated slice-by-slice (5 mm thickness). The CT attenuation values were measured and averaged (total CT attenuation value/number of slices). Renal pelvis urine attenuation was measured at mid-calyx level and bladder urine attenuation within the lumen by two independent urologists using circular regions of interest (ROIs) ≥100 mm^2^ (avoiding calyceal walls and artefacts), demonstrating substantial agreement (*κ* = 0.83, 95% CI [0.77–0.89]) [8]. All scans complied with dose audits (mean dose-length product [DLP]: 250 mGy · cm ± 10%). The renal pelvic urine volume was estimated as a quasi-ellipsoid using the following formula: volume (mL) = 0.532 × length (cm) × width (cm) × depth (cm). This geometric approximation assumes regular renal pelvic morphology and has not been formally validated in infected systems, where pathological distortions may cause volumetric underestimation.

### 2.4. Statistical analysis

Data were subjected to descriptive analyses and normality testing using SPSS version 27.0. Continuous variables were analyzed using the *t*-test, whereas categorical variables were evaluated using the chi-squared test. All clinical data were assessed using univariate logistic regression analysis. Statistically significant variables derived in univariate analyses were included in the multivariate logistic regression analysis. Statistical significance was set at *P* < 0.05. Receiver operating characteristic (ROC) curves were plotted to assess the cutoff values for statistically significant indicators. A predictive model was constructed using the statistically significant variables and nomograms. Fitting and decision curve analyses (DCAs) were used to evaluate the model efficacy and clinical utility.

This retrospective case-control study enrolled all eligible patients who met the inclusion criteria during the study period (final cohort: *n* = 249; 41 culture-positive cases and 208 culture-negative controls). Because this was a retrospective analysis, formal *a priori* power calculations were not feasible. *Post hoc* power analysis confirmed 83% power (*α* = 0.05) to detect medium effect sizes (Cohen’s *d* ≥ 0.5) given 41 cases and 208 controls. This meets the recommended thresholds for diagnostic accuracy studies and is consistent with the statistical guidelines for such studies.

## 3. Results

### 3.1. Participants selection and group allocation

In total, 491 patients who underwent surgical procedures for urinary tract stones at our hospital between January 2024 and June 2024 were enrolled in this study. Among them, 249 met the inclusion criteria. They were categorized into two groups based on the urine culture results: 208 patients with negative urine cultures in the control group and 41 patients with positive urine cultures in the infected group.

### 3.2. Microbiome distribution in urine samples from patients with positive urine cultures

In the infected group, *Enterococcus faecalis* (34.1%) and *Escherichia coli* (24.4%) were the predominant pathogens. All the isolated microbiomes were significantly associated with elevated renal pelvic urine CT attenuation values. The complete distribution of the pathogens is shown in Table 1.

**Table 1.** Microbial flora distribution.

Bacterial species	Number of cases (*n*)	Percentage (%)	Mean CT value of renal pelvic effusion (HU)
*E. faecalis*	14	34.1	13.61
*E. coli*	10	24.4	10.82
*Klebsiella pneumoniae*	3	7.3	14.45
*Streptococcus agalactiae (Group B)*	2	4.9	13.4
*Enterobacter cloacae*	2	4.9	9.65
*Proteus mirabilis*	2	4.9	12.8
*Trichosporon asahii*	1	2.4	23.2
*Acinetobacter baumannii*	1	2.4	20.7
*Streptococcus viridans*	1	2.4	8.5
*Klebsiella oxytoca*	1	2.4	14.8
*Corynebacterium jeikeium*	1	2.4	23.4
*Flavobacterium odoratum*	1	2.4	17.8
*Staphylococcus haemolyticus*	1	2.4	15.1
*Staphylococcus intermedius*	1	2.4	11.5

### 3.3. Comparisons of general information, laboratory metrics, and imaging metrics between the two groups

Patients in the infected group were significantly older *(P* < 0.0001), had a higher proportion of females (*P* = 0.001), had fewer erythrocytes (*P* = 0.009), lower hemoglobin (*P* < 0.0001), lower albumin (*P* = 0.007), higher urinary LE (*P* < 0.0001), higher urinary nitrite (*P* < 0.0001), and higher urine protein (*P* < 0.0001) levels, and had significantly higher CT attenuation values in renal pelvis urine (*P* < 0.0001) (Table 2).

**Table 2.** Baseline characteristics.

Variables	Control group (*n* = 208)	Infection group (*n* = 41)	*P*
Basic information			
Age	55.16 ± 12.7	63.85 ± 11.89	<0.001
Sex	Male 167 (80%)	Male 23 (56%)	0.001
	Female 41 (20%)	Female 18 (44%)	
Blood parameters			
CRP (mg/L)	11.43 ± 19.43	9.99 ± 19.38	0.664
WBC (×10^9^/L)	7.84 ± 2.56	7.61 ± 2.01	0.527
RBC (×10^12^/L)	4.64 ± 0.47	4.36 ± 0.61	0.009
Hb (g/L)	139.97 ± 14.23	129.42 ± 16.17	<0.001
Plt (×10⁹/L)	240.09 ± 67.67	236.90 ± 69.88	0.784
Neutrophils (×10⁹/L)	5.58 ± 2.41	5.24 ± 2.08	0.406
Lymphocytes (×10⁹/L)	1.6 ± 0.58	1.64 ± 0.59	0.734
Monocytes (×10⁹/L)	0.47 ± 0.21	0.48 ± 0.19	0.772
Alb (g/L)	43.67 ± 2.87	42.28 ± 3.54	0.007
Uric acid (μmol/L)	377.36 ± 103.25	358.36 ± 120.59	0.296
Creatinine (μmol/L)	106.63 ± 99	98.82 ± 48.57	0.623
Urine parameters			
Urine leukocyte esterase			<0.001
−	155	9	
+	36	11	
++	14	14	
+++	3	7	
Nitrite			<0.001
−	208	37	
+	0	4	
Urine protein			<0.001
−	181	25	
+	18	6	
++	8	8	
+++	1	1	
Urine glucose			0.554
−	178	36	
+	8	1	
++	9	1	
+++	7	3	
++++	6	0	
Ketones			0.602
−	204	40	
+	2	1	
++	2	0	
Urine specific gravity	1.02 ± 0.01	1.02 ± 0.01	0.391
Urine pH	6.07 ± 0.71	6.31 ± 0.89	0.058
RBC/HP	883.35 ± 4443.55	470.85 ± 1401.44	0.557
WBC/HP	69.58 ± 514.81	343.54 ± 1637.28	0.295
Imaging			
Mean CT value of ladder urine (HU)	11.46 ± 7.79	10.32 ± 7.99	0.395
Mean CT value of renal pelvis urine (HU)	5.21 ± 5.71	13.41 ± 5.11	<0.001
Volume of renal pelvic effusion (mL)	29.84 ± 45.99	32.35 ± 41.14	0.745

CRP: C-reactive protein, WBC: white blood cell, RBC: red blood cell, Hb: hemoglobin, Plt: platelet, Alb: albumin, RBC/HP: red blood cells per high-power field, WBC/HP: white blood cells per high-power field.

All patients met comorbidity-free criteria: no urinary tract abnormalities, systemic diseases, prior UTIs, or recent antibiotics. UTIs solely attributed to stone obstruction.

### 3.4. Analysis of factors associated with true bacteriuria secondary to renal and ureteral stones

Statistically significant metrics, including age, sex, erythrocytes, hemoglobin, albumin, urinary LE, urinary nitrite, urine proteins, and CT attenuation value of renal pelvis urine, were incorporated into a multivariate logistic regression analysis. The results suggested that age (*P =* 0.01), urinary LE (*P =* 0.013), and CT attenuation values of renal pelvis urine (*P < 0.0001*) were significantly associated with the urine culture results (*P* < 0.05). Specifically, advanced age, positive urinary LE, and elevated CT attenuation value of renal pelvic urine were independent risk factors for bacteriuria secondary to renal and ureteral stones (Table 3).

**Table 3.** Logistic regression analysis of factors associated with secondary true bacteriuria in patients with nephroureteral calculi.

Variables	OR	95% CI	*P*-Value
Age	1.083	1.019–1.150	0.010
Sex	0.550	0.108–2.792	0.471
RBC	3.916	0.465–33.002	0.209
Hb	0.953	0.897–1.012	0.119
Alb	1.192	0.948–1.499	0.132
Urine leukocyte esterase	2.682	1.232–5.880	0.013
Urine protein	0.962	0.284–3.261	0.950
Mean CT value of renal pelvic effusion	1.465	1.263–1.700	<0.0001

RBC: red blood cell, Hb: hemoglobin, Alb: albumin.

### 3.5. Predictive modeling of true bacteriuria secondary to stones and plotting of ROC curves

**Figure 2.**
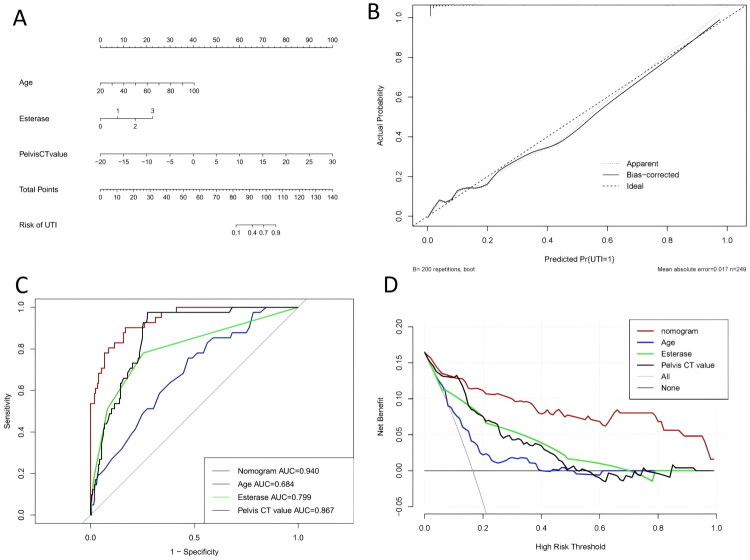
Predictive model analysis of secondary true bacteriuria in patients with nephroureteral calculi. UTI: urinary tract infection. (A) Nomogram for diagnosing clinically significant secondary UTIs in patients with hydronephrosis following urolithiasis. (B) Calibration curve of the nomogram. (C) ROC curve: comparison of the nomogram model with individual risk factors in diagnosing clinically significant secondary UTIs. (D) Clinical DCA: diagnostic performance of the nomogram model vs. individual risk factors.

A nomogram model was constructed based on three risk factors: age, urinary LE level, and CT attenuation value of renal pelvis urine. A score corresponding to each predictive variable was assigned based on line segments in the nomogram. The total score derived from the sum of these individual scores was mapped to a predicted risk, representing the probability of developing UTIs secondary to stones (Figure 2(A)). ROC curves were generated for the nomogram, and the individual risk factors of age, urinary LE level, and CT attenuation value of renal pelvic urine are presented in Figure 2(C).

The area under the curve (AUC) of the nomogram model was 0.940 (AUC: 0.940, 95% CI: 0.906–0.975); that of age was 0.684 (AUC: 0.684, 95% CI: 0.599–0.770; cutoff value: 57.5 years; sensitivity: 0.756, 95% CI: 0.634–0.878; specificity: 0.529, 95% CI: 0.456–0.596); that of urinary LE was 0.799 (AUC: 0.799, 95% CI: 0.717–0.882; cutoff value: 0.5 [1+]; sensitivity: 0.780, 95% CI: 0.659–0.802, specificity: 0.755, 95% CI: 0.688–0.801); and that of CT attenuation value of renal pelvis urine was 0.867 (AUC: 0.867, 95% CI: 0.817–0.916; cutoff value: 7.25 HU; sensitivity: 0.976, 95% CI: 0.927–1; specificity: 0.726, 95% CI: 0.668–0.783) [9]. The calibration curve of the nomogram model, as presented in Figure 2(B), suggests that it is reliable for predicting infections secondary to stones. Clinical DCA indicated substantial net benefits across a wide clinical threshold range in the nomogram model with single risk factors, including age, urinary LE, and CT attenuation value of renal pelvis urine (Figure 2(D)).

DeLong’s test revealed significantly superior diagnostic performance of the nomogram (AUC = 0.94) compared to individual predictors: age (AUC = 0.684; ΔAUC = 0.256, 95% CI: 0.178–0.334; *P* < 0.001), urinary LE (AUC = 0.799; ΔAUC = 0.141, 95% CI: 0.075–0.207; *P* < 0.001), and CT attenuation value (AUC = 0.867; ΔAUC = 0.073, 95% CI: 0.024–0.123; *P* < 0.001). These results confirmed the enhanced accuracy of the multifactorial model [10] (Table 4).

**Table 4.** Superiority of the nomogram over individual predictors: AUC comparisons using DeLong’s test.

Comparison	Nomogram AUC	Predictor AUC	AUC difference (95% CI)	*P*-Value
Nomogram vs. age	0.94	0.684	0.256 (0.178–0.334)	<0.001
Nomogram vs. urine leucocyte esterase	0.94	0.799	0.141 (0.075–0.207)	<0.001
Nomogram vs. CT value	0.94	0.867	0.073 (0.024–0.123)	<0.001

## 4. Discussion

Ureteroscopy with laser lithotripsy is the primary surgical approach for urinary tract stones. However, uncontrolled preoperative UTIs increase the risk of severe postoperative complications, including urosepsis. While UTI diagnosis primarily relies on urinalysis and culture, neither test offers both high diagnostic accuracy and timeliness. Non-contrast CT, the standard for stone diagnosis, offers potential as a rapid adjunctive tool. This study investigated the diagnostic utility of renal pelvic urine CT attenuation (HU) for true bacteriuria secondary to stones and developed a predictive nomogram integrating HU with established clinical markers. Our results demonstrate high diagnostic performance (AUC = 0.94) and clinical utility for this model.

Univariate analysis confirmed that female sex was associated with a higher UTI incidence (*P* < 0.01), likely reflecting anatomical vulnerability. However, in multivariate regression, female sex did not retain independent significance, potentially due to confounding by age and anatomical factors; instead, advanced age, positive urinary LE, and elevated renal pelvis HU emerged as independent risk factors. Advanced age aligns with the known increased incidence of stones and immunosenescence, corroborating its role as a risk factor [11].

Microbiologically, the predominance of *E. faecalis* (34.1%) and *E. coli* (24.4%) (Table 1) aligns with classic uropathogen profiles [12,13]. Crucially, HU elevation occurred universally in patients with all isolated pathogens and consistently exceeded the diagnostic threshold (HU > 7.25), regardless of the bacterial species. This indicates that the infectious burden, rather than microbial identity, drives attenuation changes, supporting the use of HU as a pathogen-agnostic screening biomarker.

Regarding rapid urinary biomarkers, nitrite positivity relies on nitrate-reducing bacteria and optimal conditions, limiting its sensitivity. Although predominant UTI pathogens here were often nitrite-positive, the significant proportion (36.6%) of non-enteric pathogens explains why nitrite was not an independent risk factor in our regression. In contrast, urinary LE, reflecting neutrophil infiltration and degranulation, was significantly higher in the infection group and emerged as a strong independent predictor (AUC = 0.799), consistent with its established high sensitivity and specificity for UTI diagnosis [14–16].

Beyond biomarkers, imaging characteristics, particularly renal pelvis HU, demonstrated significant diagnostic value for stone-associated infections. However, the lack of association observed for other factors like renal pelvic urine volume and bladder HU warrants attention. While hydronephrosis is traditionally linked to infection risk, our study found no significant volume difference between groups. This discrepancy may relate to population selection, measurement methods, or infection stage. Future studies should define the temporal relationship between obstruction duration, pelvic volume, and infection risk [17,18]. Bladder urine HU showed no difference, likely due to dilution from bilateral output and, in cases of complete obstruction, the inability of infected pelvic urine to reach the bladder [19].

The significantly elevated renal pelvic HU in infected patients primarily arises from pathological alterations in urine composition. While normal urine approximates water density (0–10 HU), infection introduces high-density elements: inflammatory exudates (pyuria, ∼30–45 HU), increased proteins (∼20–40 HU), and hematuria (∼30–45 HU) [20]. Critically, infection-associated crystalluria—driven by urease-producing bacteria alkalinizing urine and promoting precipitation—contributes substantial high-density particulate matter (often >100 HU). Fragments from infected stones may further increase attenuation, contrasting with metabolic stones where acidic urine solubilizes debris, yielding lower HU values [21–23]. Our identified cutoff (>7.25 HU) demonstrated high sensitivity (97.6%) and specificity (72.6%) for preoperative diagnosis of stone-related bacteriuria. This value exceeds Boeri’s threshold for pyonephrosis (6.3 HU) [24], and approaches their sepsis-prediction threshold (7.3 HU), yet remains lower than values reported for advanced pyonephrosis (e.g., 8–9.5 HU) [25–27]. This variance reflects differences in diagnostic targets (localized pus vs. early colonization vs. sepsis risk) and disease stage. Technical factors (ROI methodology) may also contribute to reported differences.

Literature suggests synergistic predictors beyond HU influence bacteriuria risk. These include CT markers of obstruction severity (e.g., ureteral wall thickening >2.3 mm [28], ureter-to-stone ratios [29,30], ureteral dilation promoting stasis/biofilm [5] and systemic inflammation indices (elevated neutrophil-to-lymphocyte ratio [NLR] and platelet-to-lymphocyte ratio [PLR] [31,32] reflecting barrier disruption and correlating with HU elevation). These factors compound: impacted stones with mucosal oedema/inflammation significantly elevate infection risk compared to transient obstruction. Crucially, our prediction model combining renal pelvic HU, age, and urine LE achieved superior performance (AUC = 0.94) compared to any single indicator, demonstrating significant clinical potential. However, interpretation requires caution regarding confounding comorbidities. Conditions like diabetes (glycosuria, osmotic shifts), hypertension (impaired renal defenses), dyslipidemia/obesity (neutrophil dysfunction), and advanced age (immunosenescence, mucosal atrophy) amplify infection risk through distinct pathways [33–35], complicating diagnostic correlations. Anatomically, an increased renal pelvic anteroposterior diameter may prolong stasis, facilitating colonization and sediment accumulation, potentially synergizing with HU to improve infection detection [35]. Elevated HU thus represents a composite signal reflecting the inflammatory milieu shaped by comorbidities and stone type.

This model enables rapid risk stratification for stone-associated bacteriuria using three routinely available parameters: age, urine LE, and renal pelvis HU. Clinically, it could enhance decision-making by facilitating early identification of high-risk patients in emergency settings, prompting timely empirical antibiotics guided by local susceptibility. However, clinicians must recognize inherent diagnostic risks: false positives (elevated HU without bacteriuria) may lead to unnecessary antibiotics or premature surgery; false negatives (low HU with occult infection) could delay intervention, increasing urosepsis risk in obstruction. Mandatory culture-guided therapy adjustment is essential, with minimum durations of 14–21 days, extended to 4–6 weeks if source control is delayed [36]. By translating CT-derived HU into quantifiable infection predictors, the model bridges radiology with therapeutics, potentially optimizing resource allocation (targeted cultures) and improving outcomes.

Study limitations require consideration. The nomogram was derived from patients with unilateral stones complicated by renal pelvic infection, limiting generalizability. As a single-center retrospective study of a tertiary cohort, unmeasured confounding is possible, and results may not reflect community-level UTIs. The group size disparity (controls: *n* = 208; infection: *n* = 41) reflects the lower incidence of stone-associated UTIs in our rigorously screened cohort; while potentially affecting statistical power, this imbalance is inherent to studying complicated infections. Reliance on HU across different CT scanners remains a constraint due to inherent calibration differences, necessitating future multicenter validation with harmonized equipment. Although inter-observer agreement for HU measurements was substantial (κ = 0.83), intraobserver variability was unquantified. Mandatory ROI size/positioning mitigated but did not eliminate this limitation. These factors necessitate cautious interpretation and highlight the need for prospective multicenter validation to confirm generalizability [37].

In conclusion, advanced age, urine LE, and elevated renal pelvic HU are key risk factors for bacteriuria secondary to stones. Our nomogram integrating these factors provides rapid, accurate risk assessment (AUC = 0.94) with significant clinical translation potential. Future prospective multicenter studies should establish morbidity-adjusted HU diagnostic thresholds, stratify patients by HU level to define optimized cutoffs predicting clinical evolution, evaluate HU-guided antibiotic stewardship accounting for comorbidity-specific kinetics, and integrate serial HU dynamics with novel biomarkers to develop precision management for high-risk populations.

## Data Availability

The data related to this research have not been stored in a publicly accessible database but can be obtained from the corresponding author upon request.
